# Evaluation of Subsequent Heat Treatment Routes for Near-β Forged TA15 Ti-Alloy

**DOI:** 10.3390/ma9110872

**Published:** 2016-10-26

**Authors:** Zhichao Sun, Huili Wu, He Yang

**Affiliations:** State Key Lab of Solidification Processing, Department of Materials Science and Engineering, Northwestern Polytechnical University, Xi’an 710072, China; 18729241566@163.com (H.W.); yanghe@nwpu.edu.cn (H.Y.)

**Keywords:** TA15 Ti-alloy, near-β forging, heat treatment, microstructural characteristic, mechanical properties

## Abstract

TA15 Ti-alloy is widely used to form key load-bearing components in the aerospace field, where excellent service performance is needed. Near-β forging technology provides an attractive way to form these complicated Ti-alloy components but subsequent heat treatment has a great impact on the final microstructure and mechanical properties. Therefore evaluation and determination of the heat treatment route is of particular significance. In this paper, for the near-β forged TA15 alloy, the formation and evolution of microstructures under different subsequent heat treatment routes (annealing, solution and aging, toughening and strengthening) were studied and the cooling mode after forging was also considered. Then, the type and characteristics of the obtained microstructures were discussed through quantitative metallographic analysis. The corresponding mechanical properties (tensile, impact toughness, and fracture toughness) and effects of microstructural characteristics were investigated. Finally, for a required microstructure and performance a reasonable heat treatment route was recommended. The work is of importance for the application and development of near-β forging technology.

## 1. Introduction

Ti-alloys, such as TA15, have been widely used to form key load-bearing components in the aerospace field, which are typical hard-to-form components due to the complex in shape and hard-to-deform Ti-alloys used [1]. Furthermore, in order to meet the extreme service requirements for these components, high macro-forming precision and particularly excellent mechanical properties are needed [2]. Near-β forging technology, in which the billet and die are heated to a relatively high temperature of 15–20 °C below the β transus (for TA15 alloy it is 980–985 °C) so as to reduce the material flow stress and improve the plasticity and cavity filling capacity, provides an attractive way to form these complicated Ti-alloy components [1,3].

The mechanical properties of Ti-alloy components are determined by the final microstructure, in general which consists of the primary equiaxed α phase (α_p_), secondary α phase (α_s_), and transformed β matrix [4]. The yield strength and ultimate tensile strength of Ti-6Al-4V alloy increase with the increase of the volume fraction of the equiaxed α_p_ [5], and an increase of α_p_ improves the impact toughness and plasticity within the volume fraction of 20% [3]. The volume fraction of primary equiaxed α_p_ is related to the highest heating and holding temperature in forging or subsequent heat treatment. The secondary α_s_ determines the fracture toughness, creep properties, and fatigue crack propagation behavior [6,7], thus it is very important for equipment reliability [8,9]. The increase of volume fraction and thickness of lamellar α_s_ would improve the tensile strength, creep properties, and fracture toughness [6].

However the secondary α_s_ forms mainly during cooling after forging or the subsequent heat treatment process. Depending on the nucleation positions, the α_s_ can be classified as grain boundary α (α_GB_), grain boundary Widmanstätten α (α_WGB_), which nucleated on the formed α_GB_, and intragranular Widmanstätten α (α_WI_), which nucleated in the β grains [10,11]. The formation and evolution of α_s_ are very complex due to a variety of growth behaviors [12], diverse variants and morphologies, and growth or decomposition during thermal processing [13]. The variant selection (VS) of α_s_ is through complicated interplay between the chemical driving force (alloy chemistry, cooling rate and holding temperature) and the interfacial energy between α and β grain, etc. [14]. Therefore for the near-β forged TA15 alloy, evaluating and determining the matched heat treatment route are very important for control of secondary α_s_ and equiaxed α_p_, and further to meet the required performance.

For Ti-alloy, the one-step heat treatment (annealing), dual or multiple heat treatment routes (solution and aging, toughening and strengthening) after forging were applied [1,3,6,8,9,15]. According to the role and temperature of heat treatment for Ti-alloy, two types of annealing treatments, namely high temperature annealing and low temperature annealing, are defined. High temperature annealing refers to the treatment process whose heating and holding temperature is higher than the recrystallization starting temperature of the used Ti alloy, and whose purpose is to eliminate work hardening effect. While low temperature annealing, which is used to remove stress in forgings or stabilize the microstructure, denotes the annealing process with a heating and holding temperature lower than the recrystallization starting temperature. For annealing in Ti alloy, most of the existing works focused on the one-step annealing treatment. The near-β forging was not involved before it. Moreover variations in grain size and volume fraction of equiaxed α_p_, and thickness of lamellar α_s_ were investigated by changing annealing temperature [15,16], holding time [17], and cooling modes [18,19,20,21].

Generally, solution and aging, and toughening and strengthening after forging were adopted to obtain a certain kind of microstructure in a near-α or α + β alloy. Meanwhile the functions of each step heat treatment can be performed. In recent years, the dual or multiple heat treatment routes for Ti-alloy were considered by Zhu et al. [22,23] and Sun et al. [24], but the forging before heat treatment was similarly not involved in these literatures.

By considering the nearing-β forging before subsequent heat treatments, Zhou et al. [3] obtained a tri-modal microstructure in TC11 through near-β forging combined with subsequent high temperature toughening and low temperature strengthening. The volume fraction of equiaxed α_p_ was controlled to about 10% and 15% through near-β forging and increased to about 20% during subsequent heat treatments. A certain number and aspect ratio of lamellar α_s_ originating from the decomposition of the martensite formed during water quenching after forging, were obtained during toughening, and thin Widmanstätten α precipitated from β matrix during strengthening. The toughening temperature is neither too high nor too low in the two-phase field, otherwise the lamellar α_s_ meeting the requirements in the tri-modal microstructure can not be achieved. Sun et al. [25] also got a tri-modal microstructure in TA15 alloy through near-β forging (air cooling (AC) + water quenching (WQ)) combined with solution and aging. In this method, the other two constituent phases in the tri-modal microstructure, lamellar α_s_ and transformed β matrix, were formed during solution, and the aging was only used to stabilize the microstructure. Therefore the solution temperature must be appropriate, and the aging temperature should be much lower than the recrystallization temperature. However for the TA15 alloy components after near-β forging, it has still not been resolved as to what kind of subsequent heat treatment route should be adopted.

Although the volume fraction of equiaxed α_p_ was mainly determined by near-β forging in these methods [3,25], its content, morphology and distribution vary during cooling and subsequent heat treatment. The nucleation and growth of the secondary α_s_ depend on the whole hot processing (forging, cooling, and heat treatment). In addition to the grain boundaries, intracrystalline places with high-energy defects (sub-grain boundaries, dislocations) caused by deformation provide the possible nucleation sites for α_s_, which affect not only the nucleation rate of α_s_, but also atomic migration [26,27]. The subsequent heat treatment provides the driving force for precipitation and growth of α_s_. Thus, revealing the formation and evolution of primary equiaxed α_p_ and secondary α_s_ under combined action of near-β forging and subsequent heat treat routes is the first problem to be solved.

In this paper, for the near-β forged TA15 alloy, the microstructure evolution under different heat treatment routes (annealing, solution and aging, toughening and strengthening) was studied, then the difference in microstructure and the corresponding mechanical properties were discussed. The work is of importance for determination of a reasonable heat treatment route matched with near-β forging.

## 2. Materials and Methods

### 2.1. Starting Materials

**Figure 1:**
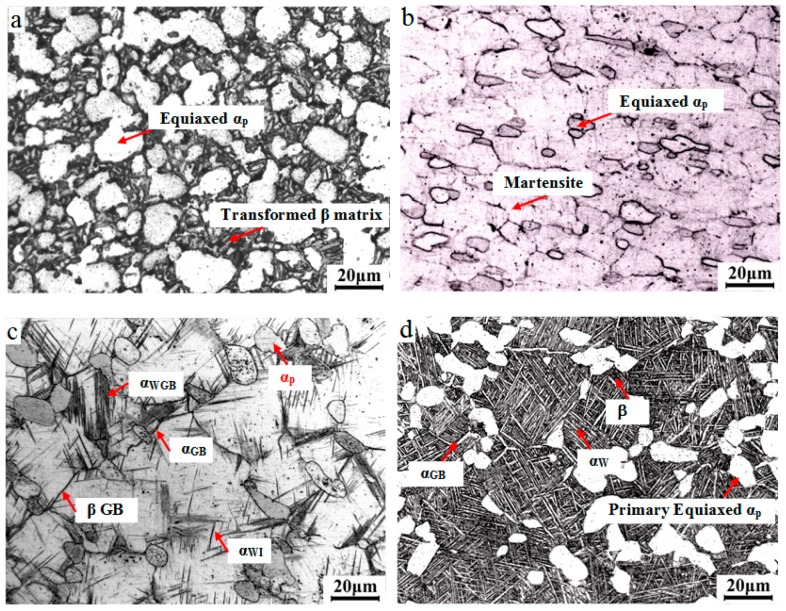
Optical micrographs of TA15 alloy after near-β forging: (**a**) Original optical micrograph of as received TA15; (**b**) 970 °C/0.1 s^−1^/65%/(WQ); (**c**) 970 °C/0.1 s^−1^/65%/(AC + WQ); (**d**) 970 °C/65%/0.1 s^−1^/(AC). WQ: water quenching, AC: air cooling.

The TA15 Ti-alloy used in the experiments was from Western Superconducting Technologies Inc. (Xi’an, China). The chemical composition of the as-received material with a β transus temperature of 980–985 °C is listed in Table 1, and its microstructure consists of about 60% (volume fraction was measured by the area fraction of the equiaxed α_p_ in microstructure) equiaxed α_p_, with an average diameter of 11 μm (average length of lines passing through the centroid of α_p_ per 2 degrees), and residual transformed β matrix, as shown in Figure 1a. The blank was processed into cylindrical specimens of Ф10 mm × 15 mm with 0.2 mm-deep shallow slots on the ends to store glass lubricant.

### 2.2. Experimental Procedure

Before near-β forging, the specimens were heated to 970 °C at a heating rate of 5 °C/s and held to obtain a homogeneous microstructure with a holding time of 5 min/mm in diameter. According to the actual forging production, a strain rate of 0.1 s^−1^ was selected and the reduction of height was 40%, 60%, or 65%. After the forging, three kinds of cooling methods were adopted, air cooling (AC), water quenching (WQ), and a short time of AC combined with WQ (AC + WQ, air cooled to about 835 °C and then water quenched), which was used to consider the temperature drop during forging transfer—the forgings were transferred from press to water tank for quenching. Three kinds of subsequent heat treatment routes, annealing (low-temperature annealing, LTA, high-temperature anneal, HTA), solution and aging (SA), and toughening and strengthening (TS) were considered; a detailed experiment scheme is given in Table 2. During subsequent heat treatment the same heating rate was adopted.

After the experiment for the specimens the metallographic observation was implemented by an optical microscope (Leica, Wetzlar, Germany) and quantitative metallographic analysis was carried out using an Image-Pro Plus 6.0 image analysis system (Media Cybernetics, Silver Spring, MD, USA, 2006). Meanwhile the typical mechanical properties required by aeronautic forgings, such as room and high temperature tensile properties, impact toughness, and fracture toughness, were measured according to the industry standards—“Metal test method”, HB5143-80, HB5195-81, HB5144-80, HB5142-80 [28], respectively.

## 3. Results and Discussion

### 3.1. Microstructural Evolution of Near β-forged TA15 Alloy under Different Heat Treatment Routes

Figure 1 shows the optical micrographs of TA15 alloy before (Figure 1a) and after near-β forging (970 °C/0.1 s^−1^/65%) and cooled by different modes (Figure 1b–d). The water quenched microstructure in Figure 1b (water quenching after near-β forging) exhibits about 13.5% equiaxed α_p_ and residual martensite. A high cooling rate (higher than the critical cooling rate) resulted in martensite transformation and the reservation of the distortion energy and defects caused by forging. Air cooled for a short time and then water quenched after near-β forging, the microstructure, which is shown in Figure 1c, consists of equiaxed α_p_, grain boundary α (α_GB_), grain boundary Widmanstätten α (α_WGB_), intragranular Widmanstätten α (α_WI_) and residual martensite. β→α phase transformation occurred at a low cooling rate, and the precipitated α phase preferred to nucleate at β grain boundaries, then on the formed α_GB_ and defects in β grains [20]. Only part of β transformed into α phase during the short time of air cooling, and the residual β phase converted to martensite due to a high cooling rate. A heterogeneous precipitation of α_WGB_ within the same or different β grains can be observed in Figure 1c, which results from both the variation selection (VS) of the α_GB_ and α_WGB_ [14]. When air cooled, the microstructure (Figure 1d) shows a typical bimodal microstructure, a large amount of very thin α_W_ (α_WGB_ and α_WI_) with an interlaced distribution, a small amount of equiaxed α_p_ and residual β, and very few α_GB_. Since there existed lots of defects in the β grains after forging, the α_WI_ was the main component of secondary α_s_ in Figure 1d.

**Figure 2:**
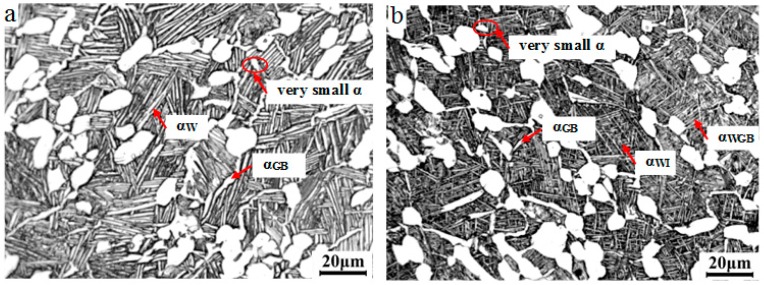
Optical micrographs of TA15 alloy after near-β forging and low-temperature annealing: (**a**) 970 °C/0.1 s^−1^/40%/AC + 830 °C/1 h/AC; (**b**) 970 °C/0.1 s^−1^/40%/WQ + 830 °C/1 h/AC.

The optical micrographs of TA15 alloy after near-β forging (AC or WQ) and subsequent low-temperature annealing (LTA) (830 °C/1 h/AC) are exhibited in Figure 2. In general they had the same microstructural type as that in Figure 1d (near-β forging/AC), i.e., one step subsequent low-temperature annealing did not change the microstructural type that was obtained by near-β forging/AC. Seldom very small equiaxed α (marked by red ellipse in Figure 2) appeared due to globularization of α_GB_ or static recrystallization of α_p_ [13]. When air cooled after forging and annealing, the relatively long and thick clustered α_W_ appeared, as shown in Figure 2a. Forged and air cooled (Figure 1d) clustered α_W_ preferentially nucleated and grew on the existing α_GB_ due to a low supercooling degree [20], meanwhile most of the distortion energy caused by forging was consumed by the precipitation and growth of the α_W_ during air cooling after forging. When annealed, some formed α_W_ (little ones) dissolved, while some grew both in length and width direction during heating and holding. In succedent AC, the existing α_W_ thickened a bit, and it already had lots of α phase (α_W_ and α_p_) which consumed a large amount of α-stablized elements, hence only seldom α_W_ was formed. Short and thin disordered α_W_ presented in Figure 2b after near-β forging (WQ) combined with LTA. The distortion energy and defects kept by water quenching provided a large driving force and enough possible sites for nucleation and growth of α_W_ in subsequent heat treatment [26]. Moreover they promote more variants of α_W_ [14]. As a result lots of thin α_W_ precipitated both on formed α_GB_ (α_WGB_) and inside β grains (α_WI_) in a disordered manner during subsequent annealing.

**Figure 3:**
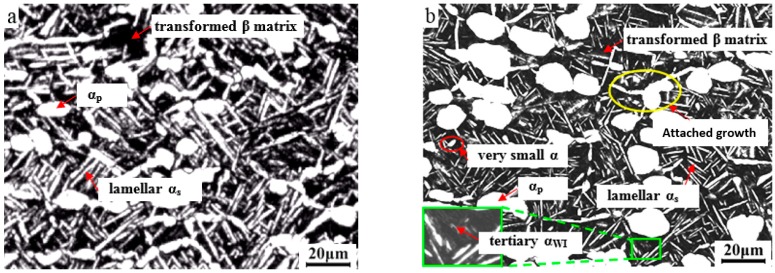

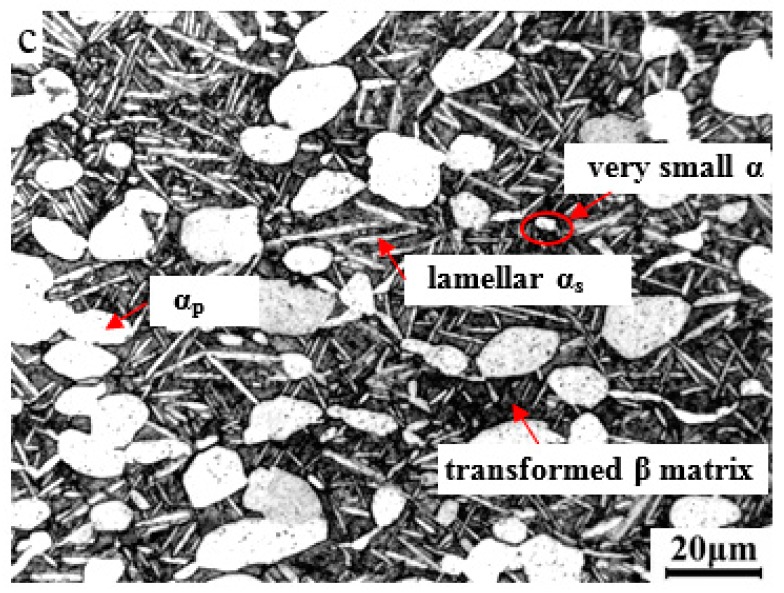
Optical micrographs of TA15 alloy after near-β forging and solution + aging treatment: (**a**) 970 °C/0.1 s^−1^/65%/WQ + 930 °C/1 h/AC + 550/5 h/AC; (**b**) 970 °C/0.1 s^−1^/60%/(AC + WQ) + 930 °C/1 h/AC + 550 °C/5 h/AC; (**c**) 970 °C/0.1 s^−1^/60%/(AC + WQ) + 930 °C/1 h/AC.

Figure 3a,b display the optical micrographs of TA15 alloy through near-β forging with a cooling mode of WQ or AC + WQ combined solution + aging (SA, 930 °C/1 h/AC + 550 °C/5 h/AC). For the near-β forged (970 °C/0.1 s^−1^/65%/WQ) specimen, the typical tri-modal microstructure was obtained (Figure 3a) after SA (930 °C/1 h/AC + 550 °C/5 h/AC), consisting of small equiaxed α_p_, thick and long secondary lamellar α_s_, and transformed β matrix. Its formation process was as follows. When heated at 930 °C after forging, the existing martensite decomposed to form secondary α_s_ and β phases. A larger amount of α_s_ nucleated at β grain boundaries (GBs) and in β grains simultaneously and grew rapidly. As holding time was prolonged, some thin α_s_ dissolved and others thickened through merging [29]. In subsequent AC, the undissolved α_s_ thickened further, and tertiary lamellar α_s_ precipitated from β to form the transformed β matrix.

A quasi tri-modal microstructure, including coarse equiaxed α_p_, scattered thick lamellar α_s_, and very thin tertiary α_WI_, was obtained after near-β forging (AC + WQ, air cooled for a short time and then water quenched) and SA treatment, as shown in Figure 3b. Figure 3c illustrates the optical micrograph without subsequent aging. When heated and held at 930 °C the driving force for nucleation of α_s_, which originated from the decomposition of the martensite, was insufficient because it was consumed partially during the short time of AC after forging, and the formed α_s_ thickened and lengthened. For equiaxed α_p_, on one hand the growth of already-nucleated grains, meta-dynamic and static recrystallization of α_p_ occurred and resulted in very small α, as marked by red ellipses in Figure 3b,c [25]. On the other hand the existing α_p_ coarsened through the Ostwald ripening effect or attached-growth of α phase (as marked by the yellow ellipse in Figure 3b) [30]. It can also be seen that for near-β forged (AC + WQ) TA15 alloy, the followed low-temperature aging treatments (550 °C/5 h/AC) resulted in little variation in the microstructure only after solution treatment (930 °C/1 h/AC). However it should be noted that the aging treatment is important for stability and homogenization of the microstructure.

**Figure 4:**
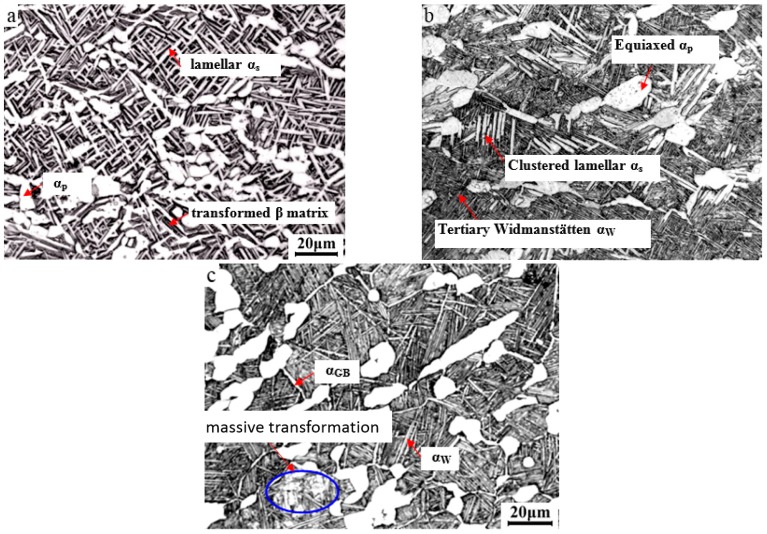
Optical micrographs of TA15 alloy after near-β forging and toughening + strengthening treatment: (**a**) 970 °C/0.1 s^−1^/65%/WQ + 950 °C/100 min/WQ + 800 °C/8 h/AC; (**b**) 970 °C/0.1 s^−1^/60%/(AC + WQ) + 950 °C/1 h/WQ + 800 °C/5 h/AC; (**c**) 965 °C/0.1 s^−1^/65%/AC + 970 °C/1.5 h/WQ + 850 °C/1.5 h/AC.

Figure 4 presents the optical micrographs of TA15 alloy after near-β forging and toughening + strengthening (TS). After 970 °C/0.1 s^−1^/65%/WQ + 950 °C/100 min/WQ + 800 °C/8 h/AC, the microstructural type was a typical tri-modal microstructure (Figure 4a). Compared with the obtained microstructure after solution and aging (Figure 3a), more short and disordered secondary lamellar α_s_ appeared and thin interlaced tertiary α_w_ on the β matrix showed a scattered distribution. This was due to two times of water quenching and a higher holding temperature of 950 °C. Heated and held at 950 °C disordered lamellar α_s_ formed through decomposition of martensite, which resulted from WQ after forging. Meanwhile some small equiaxed α_p_ formed through recrystallization. Water quenched again after heating at 950 °C for 100 min, the high-temperature β transformed into martensite once more. In the subsequent strengthening treatment (800 °C/8 h), the martensite decomposed to form α_s_ and β phase. Some scattered thin interlaced tertiary α_W_ formed through β→α phase transformation during air cooling [30]. Due to rapid WQ after the toughening temperature (950 °C), growing and coarsening of the equiaxed α_p_ were restrained. During the subsequent strengthening process, due to a relative low temperature (800 °C) the driving force for the equiaxed α_p_ coarsening was insufficient, which resulted in small equiaxed α_p_.

Figure 4b shows the optical micrograph after 970 °C/0.1 s^−1^/60%/(AC + WQ) + 950 °C/1 h/WQ + 800 °C/5 h/AC. Compared with that in Figure 4a, a short time of AC was involved but the microstructure showed a great difference. Less clustered lamellar α_s_ were obtained but a large amount of thin tertiary Widmanstätten α_W_ appeared. Meanwhile the equiaxed α_p_ was coarse. The main reason was that short AC before WQ after forging changed the formation stage and source of lamellar α_s_. For near-β forged (AC + WQ) specimen, the final lamellar α_s_ came from the residual Widmanstätten α_W_ formed during AC and decomposition of residual martensite during high temperature toughening, but for near-β forged (WQ) specimen, it only came from the decomposition of martensite.

When the toughening temperature was equal or higher than the forging temperature, an interesting phenomenon was discovered. For TA15 alloy after 965 °C/0.1 s^−1^/65%/AC + 970 °C/1.5 h/WQ + 850 °C/1.5 h/AC (Figure 4c), a bimodal microstructure was obtained and it was similar to that as shown in Figure 1d. However the Widmanstätten α_W_ were thicker and longer and part of them showed a clustered distribution (Figure 4c). The residual α_GB_ distributed along the contour of β GBs. After near-β forging (AC), about 20% equiaxed α_p_ and a large amount of Widmanstätten α_W_ appeared (Figure 1d). Held at 970 °C for enough time (1.5 h), the existing Widmanstätten α_W_ dissolved into β phase and the distortion energy and defects caused by forging were consumed. Water quenched supercooled martensite was obtained and when heated and held at 850 °C, martensite decomposed into α and β phases. Due to the low transformation temperature and inadequate driving force (cooling rate), the Widmanstätten α_W_ nucleated on β GBs and α/β interface preferentially and grew into a clustered morphology. Meanwhile for martensite the massive transformation, which may have been caused by the composition inhomogeneity of the material, occurred as marked by the blue ellipse in Figure 4c.

### 3.2. Microstructural Characteristics and Mechanical Properties

**Figure 5:**
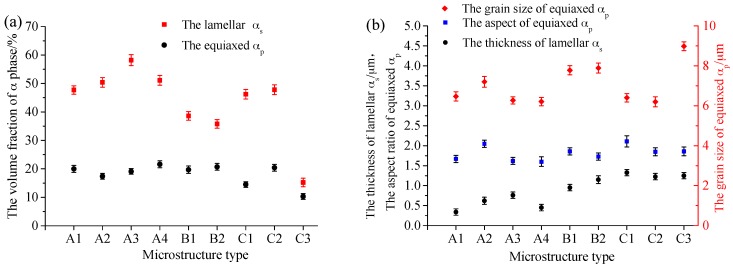
Measured microstructural characteristic parameters obtained microstructures in TA15 alloy: (**a**) volume fraction of α phase; (**b**) size of α phase.

**Figure 6:**
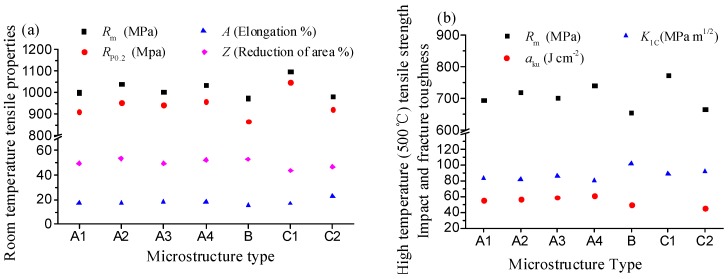
Mechanical properties of TA15 alloy with different microstructures: (**a**) Room-temperature tensile properties; (**b**) high-temperature (500 °C) tensile strength, impact, and fracture toughness.

Based on the analysis above, for the near-β forged TA15 alloy after the heat treatment routes involved, three types of microstructures were obtained, which were bimodal (Figure 1d, Figure 2a,b and Figure 4c), quasi-tri-modal (Figure 3b,c) and tri-modal (Figure 3a and Figure 4a), defined as type A, B, and C, respectively. Comparison of the characteristics of the obtained microstructures and constituent phases and corresponding processing routes are presented in Table 2. Figure 5 shows the measured microstructural characteristic parameters and Figure 6 shows the corresponding mechanical properties.

For TA15 alloy the bimodal microstructures were obtained through near-β forging and AC (Figure 1d, type A1 in Table 2), near-β forging + LTA (Figure 2, types A3 and A4 in Table 2), and near-β forging + NTS (near-β toughening and strengthening) (Figure 4c, type A2 in Table 2), respectively. The volume fraction of primary equiaxed α_p_ changed between 18.2% and 21.6%, and average grain size (diameter) between 6.26 and 7.20 μm (Figure 5). The volume fraction of the Widmanstätten α_W_ changed between 47.7% and 58.2%, but its distribution and thickness showed an obvious difference. For microstructure of type A1, there existed a great deal of very thin interlaced α_W_ with a thickness of about 0.35 μm appearing, and spreading all over the matrix (Figure 1d, near-β forging/AC). For type A4 (Figure 2b, near-β forging/WQ + LTA) thin interlaced α_W_ were obtained (0.45 μm). For type A3 (Figure 2a, near-β forging/AC + LTA) the α_W_ was relatively thick, 0.75 μm, and showed a clustered distribution. For type A2 (Figure 4c, near-β forging/AC + NTS) long and thin clustered α_W_ appeared.

The quasi tri-modal microstructures were obtained through near-β forging (AC + WQ) combined with SA (Figure 3b, type B2 in Table 2) or HTA (Figure 3c, type B1 in Table 2), consisting of large primary equiaxed α_p_, short and thick lamellar α_s_, and transformed β matrix. The equiaxed α_p_ had a volume fraction of 19.7%–25.4%, and an average grain size of 7.78–8.26 μm. The lamellar α_s_ had a volume fraction of 35.8%–38.6% and a thickness of 0.95–1.15 μm (Figure 5).

Tri-modal microstructures were obtained after near-β forging (WQ) and SA (Figure 3a, type C1 in Table 2), and after near-β forging (WQ) and TS (Figure 4a, type C2 in Table 2), consisting of 14.5%–20.4% small equiaxed α_p_ (6.20–6.40 μm) and 46.2%–47.8% thick lamellar α_s_ (1.20–1.35 μm) (Figure 5). For microstructure of type C2 (near-β forging/WQ + TS), thinner (1.23 μm) and interlaced lamellar α_s_ appeared but for type C1 (near-β forging/WQ + SA) α_s_ was thicker (1.33 μm) and longer (Figure 5). For type C3 (Figure 4b, near-β forging/(AC + WQ) + TS) the equiaxed α_p_ was large (8.98 μm) and there were about 15.2% moderately thick lamellar α_s_ (Figure 5), but a large amount of very thin tertiary Widmanstätten α_W_ appeared.

Bimodal microstructures of types A1–A4 possessed moderate room-temperature tensile strength (*R_m_* = 1000.0–1040.0 MPa, *R_p_*_0.2_ = 910.0–960.0 MPa) and plasticity (*A* = 17.0%–185%, *Z* = 49.5%–53.0%). As shown in Figure 6a, the tri-modal microstructure of type C1 (Figure 3a) exhibited the highest room-temperature tensile strength (*R_m_* = 1098.0 MPa, *R_p_*_0.2_ = 1049.0 MPa). G. Lutjering [31] studied the influence of processing on the microstructure and mechanical properties of (α + β) titanium alloys and found that α colony size was an important parameter for yield stress, high cycle fatigue (HCF) strength and low cycle fatigue (LCF) strength since it determined the effective slip length. In tri-modal microstructure C1 consisting of long and thick disordered lamellar α_s_, the effective slip length decrease to the width of a single lamellae plate, which is benefit for the properties aforementioned. Meanwhile ductility is improved with the increase of equiaxed α_p_ content within 20% [30]. From Figure 5a, except microstructure of type C3, type C1 had the lowest content of equiaxed α_p_ (14.5%), therefore, it possessed the lowest plasticity (*A* = 16.8%, *Z* = 43.8%). Research showed that equiaxed α_p_ contents above 20% contributed little to the increase of ductility, but had detrimental effects on other performances [32]. Thus, the tri-modal microstructure of type C2 (Figure 4a) possessed low room-temperature strength (*R_m_* = 981.3 MPa, *R_p_*_0.2_ = 822.0 MPa) but the highest elongation (*A* = 23%). Quasi tri-modal microstructure of type B (Figure 3b,c) possessed the lowest room-temperature strength (*R_m_* = 974.0 MPa, *R_p_*_0.2_ = 866.0 MPa) but the highest percentage reduction of area (*Z* = 53%). On the one hand disordered lamellar α_s_ contained in this kind of microstructure is good for the decrease of the α colony size, on the other hand a large amount of transformed β matrix increase the effective slip length. The interactions of these two effects led to the lowest room-temperature strength.

As shown in Figure 6b, for aforementioned types of microstructures their high temperature (500 °C) tensile strength has a similar variation trend as that at room temperature. Microstructure of types C1 and C2 exhibited moderate fracture toughness (*K*_1C_ = 88.6–91.5 MPa·m^1/2^) but type C2 possessed a low impact toughness (*a*_ku_ = 45.0 J·cm^−2^). Microstructures of type B possessed the highest fracture toughness (*K*_1C_ = 101.3 MPa·m^1/2^) but low impact toughness (*a*_ku_ = 49.0 J·cm^−2^). Types A1–A4 had moderate fracture toughness (*K*_1C_ = 80.8–85.5 MPa·m^1/2^) and impact toughness (*a*_ku_ = 55.4–60.0 J·cm^−2^). This is because the fracture toughness increased with the increase of the effective slip length [29].

**Figure 7:**
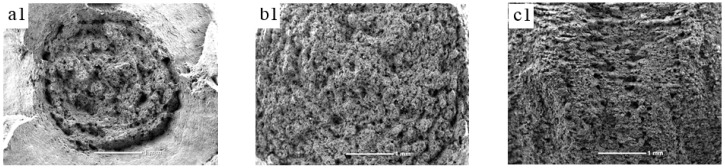

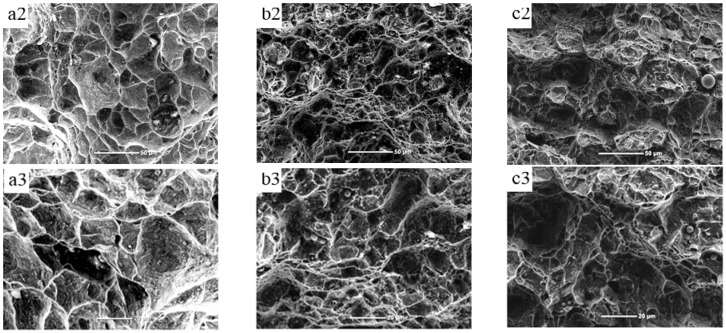
Scanning electron microscope (SEM) images of fracture with different magnifications for TA15 alloy after (**a1**) high temperature tensile test (×27); (**a2**) high temperature tensile test (×500); (**a3**) high temperature tensile test (×1000); (**b1**) room temperature tensile test (×27); (**b2**) room temperature tensile test (×500); (**b3**) room temperature tensile test (×1000); (**c1**) impact test (×27); (**c2**) impact test (×500); (**c3**) impact test (×1000).

Figure 7 shows the scanning electron microscope (SEM) images of the fractures for TA15 alloy with a tri-modal microstructure (heat treated at a condition of 975 °C/2 h/WQ + 930 °C/3 h/AC) after high temperature tensile, room temperature tensile and impact tests. From Figure 7a1,b1, both of them display the features of ductile fracture. Figure 7c1 shows aligned fibers which is the typical characteristic of an impact fracture. When the magnification increased to 500, dimples can be observed in three figures (Figure 7a2, b2 and c2). However, the dimples are different in size, depth, and number, which can also be verified by figures of a bigger magnification. Obviously, the fracture after the high temperature tensile test possessed the deepest dimples, the next was the fracture after the room temperature tensile test, and the last was the fracture after the impact test. Thus, the material possessed a high ductility at high temperature.

### 3.3. Evaluation of Heat Treatment Routes for Near-Forged TA15 Alloy

From the analysis above, for the near-β forged TA15 alloy after subsequent low-temperature annealing (LTA) and near-β toughening + strengthening (NTS), bimodal microstructures were obtained, which possessed moderated comprehensive properties (low and high temperature tensile properties, fracture and impact toughness), types A2–A4 in Figure 6. For near-β forged (AC) specimen although subsequent NTS resulted in a higher room-temperature tensile strength than LTA, about 37.5 MPa (type A2 and A3 in Figure 6a), the NTS included a near-β heat treatment (WQ) and a LTA treatment, which was more complex. Therefore the NTS was not recommended compared with LTA. For the given subsequent LTA treatment, WQ after forging resulted in thin interlaced α_W_ in the microstructure (Figure 2b), which increased the effective slip length. Thus the tensile strength could be enhanced to a certain degree (type A4 in Figure 6).

For near-β forged (WQ) specimen the subsequent SA treatment could result in a tri-modal microstructure (Figure 3a) and excellent comprehensive performance (type C1 in Figure 6), which is recommended when extreme service performance is required.

When the near-β forged specimen was air cooled for a short time and then water quenched, the subsequent SA resulted in a quasi tri-modal microstructure (Figure 3b) and high fracture toughness (type B in Figure 6b). This was similar to the microstructure after near-β forging (AC + WQ) combined with high-temperature annealing (HTA) (Figure 3c). Under this circumstance HTA was preferred.

For near-β forged (WQ) specimen subsequent TS treatment could also result in a tri-modal microstructure (Figure 4a) and a slight lower performance than that by SA. Meanwhile in the TS process the specimen was water quenched after toughening (e.g., 950 °C/100 min/WQ), this increased the possibility of shape distortion of the component due to a large degree of supercooling in WQ, especially for large complex components [33].

## 4. Conclusions

(1) Bimodal microstructure, possessing moderate low and high temperature tensile properties, fracture and impact toughness, was obtained through near-β forging combined with low-temperature annealing (830 °C/1 h/AC) or near-β toughening and strengthening treatment (970 °C/1.5 h/WQ + 850 °C/1.5 h/AC). Air cooling after near-β forging resulted in thicker (0.75 μm) clustered α_W_, while water quenching led to thin (0.45 μm) interlaced α_W_.
(2) After near-β forging (AC + WQ) combined with solution + aging (930 °C/1 h/AC + 550 °C/5 h/AC) or high-temperature annealing (930 °C/1 h/AC), a quasi tri-modal microstructure with low room and high temperature tensile strength and impact toughness but high fracture toughness was obtained.
(3) A tri-modal microstructure was obtained after near-β forging (WQ) and solution + aging (930 °C/1 h/AC + 550 °C/5 h/AC), or after near-β forging (WQ) and toughening + strengthening (950 °C/100 min/WQ + 800 °C/8 h/AC), consisting of small equiaxed α_p_ (6.20–6.40 μm) and thick lamellar α_s_ (1.20–1.35 μm), possessing good comprehensive performance matching.
(4) For near-β forged TA15 alloy, when moderate mechanical properties were required, water quenching after near-β forging and subsequent low-temperature annealing were recommended; when excellent comprehensive performance was needed, solution and aging treatment was recommended; and when high fracture toughness was considered, high-temperature annealing treatment was recommended.

## Figures and Tables

**Table 1:** Chemical composition of TA15 Ti-alloy.

Element	Al	Zr	Mo	V	Si	C	Fe	O	N	H	Ti
Nominal composition/(wt %)	6.63–6.75	2.23–2.27	1.73–1.80	2.24–2.27	<0.04	<0.006	0.14	0.12	<0.002	0.002	Balance

**Table 2:** Experimental scheme and microstructural characteristics.

No.	Forging Conditions	Cooling Mode	Heat Treatment Route	Heat Treatment Condition	Microstructural Type	Microstructural Characteristic
1	970 °C/0.1 s^−1^/65%	WQ	-	-	-	-
2	970 °C/0.1 s^−1^/65%	AC + WQ	-	-	-	-
3	970 °C/0.1 s^−1^/65%	AC	-	-	Bimodal (A1)	Very thin interlaced α_W_, small α_p_
4	970 °C/0.1 s^−1^/40%	AC	LTA	830 °C/1 h/AC	Bimodal (A3)	Thick clustered α_W_, smaller α_p_
5	970 °C/0.1 s^−1^/40%	WQ	LTA	830 °C/1 h/AC	Bimodal (A4)	Thin interlaced α_W_, smaller α_p_
6	970 °C/0.1 s^−1^/60%	AC + WQ	HTA	930 °C/1 h/AC	Quasi tri-modal (B1)	Short and thick α_s_, large α_p_
7	970 °C/0.1 s^−1^/65%	WQ	SA	930 °C/1 h/AC + 550 °C/5 h/AC	Tri-modal (C1)	Long and very thick α_s_, small α_p_
8	970 °C/0.1 s^−1^/60%	AC + WQ	SA	930 °C/1 h/AC + 550 °C/5 h/AC	Quasi tri-modal (B2)	Short and thick α_s_, large α_p_
9	970 °C/0.1 s^−1^/65%	WQ	TS	950 °C/100 min/WQ + 800 °C/8 h/AC	Tri-modal (C2)	Short and thicker α_s_, smaller α_p_
10	970 °C/0.1 s^−1^/60%	AC + WQ	TS	950 °C/1 h/WQ + 800 °C/5 h/AC	Tri-modal (C3)	Short and thicker clustered α_s_, very large α_p_
11	965 °C/0.1 s^−1^/65%	AC	NTS	970 °C/1.5 h/WQ + 850 °C/1.5 h/AC	Bimodal (A2)	Thin and long clustered α_W_, moderate α_p_

WQ—Water quenching, AC—Air cooling, LTA—Low-temperature annealing, HTA—High-temperature annealing, SA—Solution and aging, TS—toughening and strengthening, NTS—Near-β toughening and strengthening.
